# Genome-Wide Identification and Analysis Uncovers the Potential Role of JAZ and MYC Families in Potato under Abiotic Stress

**DOI:** 10.3390/ijms24076706

**Published:** 2023-04-04

**Authors:** Shan Wang, Yongbin Wang, Rui Yang, Wanhua Cai, Yaning Liu, Duanrong Zhou, Li Meng, Ping Wang, Binquan Huang

**Affiliations:** 1 State Key Laboratory for Conservation and Utilization of Bio-Resources in Yunnan, School of Agriculture, Yunnan University, Kunming 650504, China; 2 Southwest United Graduate School, Kunming 650504, China

**Keywords:** JAZ family, MYC family, potato, protein interaction, abiotic stress

## Abstract

As key regulators of the Jasmonates (JAs) signal transduction pathway, JAZ protein, and MYC transcription factors are imperative for plant response to external environmental changes, growth, and development. In this study, 18 *StJAZs* and 12 *StMYCs* were identified in potatoes. Their chromosomal position, phylogenetic development, gene structure, and promoter cis-acting parts of the *StJAZ* genes were analyzed. In addition, Protein–Protein Interaction (PPI) network analysis of *StJAZ* and *StMYC* gene families and yeast two-hybrid assay demonstrated that five StMYCs can interact with 16 StJAZs, which provides new insights into the operation mechanism of StJAZs and StMYCs in JA signal response. Moreover, we explored the expression profiles of StJAZs and StMYCs genes in different tissues and during abiotic stresses by RNA-seq data. Based on the PPI network and transcriptome data, the genes *StJAZ11*, *StJAZ16*, and *StMYC6* were chosen for further qRT-PCR study under salt or mannitol treatment. Under mannitol-induced drought or salinity treatment, the expression patterns of *StMYC6*, *StJAZ11*, and *StJAZ16* were different, indicating that the JAZ protein and MYC transcription factor may be engaged in the response of potatoes to abiotic stress, which opened up a new research direction for the genetic improvement of potatoes in response to environmental stress.

## 1. Introduction

Potatoes (*Solanum tuberosum* L.) are one of the most significant food crops in the world, which are essential for guaranteeing food security [1]. Many studies have demonstrated that plant hormones, including jasmonic acid (JA), are crucial for all phases of potato life [2,3,4,5,6,7,8]. Someone found that external administration of JA promoted the growth and differentiation of hooked apex stolon (phase I) and initial swelling stolon (phase II) cells [3]. In addition, different concentrations of JA externally applied also affected JA tuber development, mostly in the form of low-concentration promotion and high-concentration inhibition [5]. Exogenous JA plays a protective role when potatoes are stressed and partially abolished the negative effects of adversity [6].

The protein JASMONATE ZIM DOMAIN (JAZ) is essential for JA signaling. When there are few reactive JAs, the JAZ protein acts as a negative regulator and prevents the expression of *JA* response genes [9,10]. As the amount of reactive JAs rises, JA-lle-mediated JAZ proteins bind to JAs receptors and are thus degraded by the 26S proteasome. Therefore, many transcription factors (TFs), including MYC2, are released to trigger downstream gene expression [11,12]. MYC2 is the major regulatory factor in the JA signaling pathway, which can differentially regulate JA’s response to pathogens, pest resistance, wound response, and other functions [13]. The JAZ protein was found to be a direct receptor of COI1, and the MYC2 TF and the JAZ protein were implicated in the feedback regulation of the jasmonate reaction [14]. Afterward, in *Arabidopsis thaliana*, both TFs MYC3 and MYC4 can bind with JAZ protein, thereby co-activating JAs-related responses with MYC2 [15]. Subsequent research showed that a total of three MYC TFs from MYC2 to MYC4 were implicated in regulating plant growth and responding to stresses and played important roles in the JAs signaling pathway [16,17,18,19]. This clearly indicates that JAZ proteins and MYC TFs are the primary regulatory elements in the JAs signaling pathway.

In this study, the *JAZ* and *MYC* gene families in potatoes were extensively identified using genomic resources. In the meanwhile, research was conducted on phylogeny, chromosomal localization, evolutionary relationship, and gene structure. To explore the linkage between the two gene families in the process of JA signaling, we conducted promoter analysis of JAZ members and obtained the protein interaction network between *JAZs* and *MYCs* gene. Furthermore, we analyzed the patterns of *JAZs* and *MYCs* gene expression under various tissue or abiotic stresses and speculated about their role in potato growth and development or resistance to stress. This study is a reference for elucidating the function and relationship between MYC and JAZ in potatoes.

## 2. Results

### 2.1. Identification and Chromosome Mapping of StJAZ and Members of Family in Potato

Based on the potato genomic data, 18 *JAZ* genes and 12 *MYC* genes in potatoes were finally obtained, which were named *StJAZ1-StJAZ18* and *StMYC1-StMYC12*, based on their location on the chromosome (Supplementary Tables S1 and S2). The 18 *StJAZ* and 12 *StMYC* genes sequences were analyzed (Table 1 and Table 2). The aa of 18 StJAZ proteins ranged from 107 to 391, and between 144 and 701 for 12 StMYC proteins. The pI values of 18 StJAZ proteins ranged from 4.93 to 9.81, and from 5.47 to 7.61 for 12 StMYC proteins. The MW of 18 StJAZ proteins varied from 13.20 to 41.19 kDa, and from 44.08 to 75.94 kDa for 12 StMYC proteins. The 18 StJAZ and 12 StMYC proteins were predicted to be localized in the nucleus.

**Figure 1:**
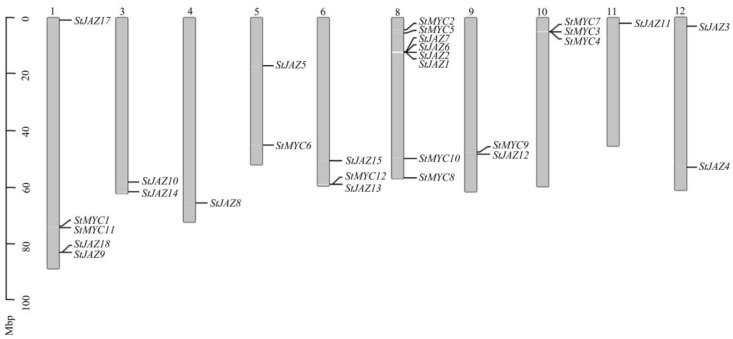
Genomic distributions of *StJAZ* and *StMYC* genes on 10 potato chromosomes (chromosomes are represented by bars, with chromosome numbers displayed at the top of the bars and gene names listed on the right. The relative chromosomal position of each gene is represented by the unit Mbp and marked on the black line on the left).

On 10 chromosomes, there were unequal distributions of the 18 *StJAZ* and 12 *StMYC* genes. (Figure 1). The five chromosomes (chromosomes 1, 5, 6, 8, and 9) contained different members of *StJAZ* and *StMYC* gene families; the four chromosomes (chromosomes 3, 4, 11, and 12) contained only the StJAZ members; and one chromosome (chromosome 10) contained only the StMYC members. There are the most genes on chromosome 8, and chromosomes 4 and 11 both contain only one gene. Taken together, all these data provide a platform for further describing JAZ and MYC family proteins or related networks in potatoes.

### 2.2. Phylogenetic Analysis of the JAZ and MYC Gene Families

**Figure 2:**
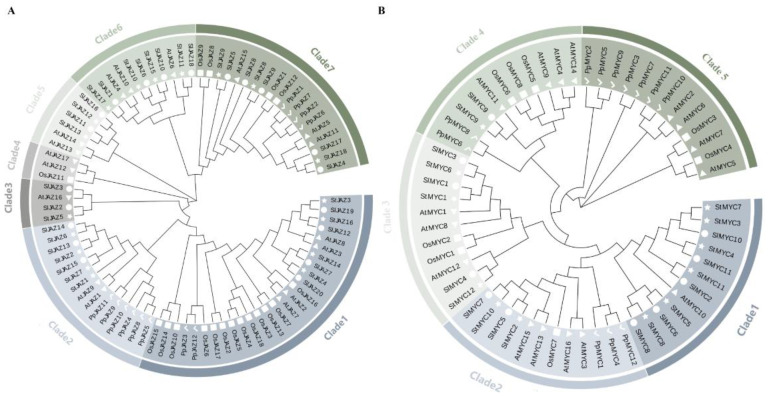
Phylogenetic tree of potato, tomato, rice, Arabidopsis, and physcomitrella patens JAZs and MYCs. (**A**) The Phylogenetic tree of JAZs; (**B**) The Phylogenetic tree of MYCs. Circles represent tomato; the squares represent rice; the triangle represents Arabidopsis; the hooks represent physcomitrella patens; and the stars represent potato.

Using MEGA X 11.0, an NJ phylogenetic tree was created for the 18 StJAZs, 18 AtJAZs, 12 PpJAZs, 18OsJAZs, and 20 SlJAZs proteins to study the evolutionary and phylogenetic links of the *JAZ* family genes between potato and the other four species. They spontaneously split off into seven sub-groups (Figure 2A). Twenty-seven JAZ proteins were in Clade1, which had the maximal number of OsJAZ. Seventeen protein members were in Clade2, and it had the most PpJAZ protein. Four JAZ proteins were in Clade3, of which two had SlJAZs:1 StJAZ, and 1 AtJAZ. There is only one OsJAZ and two AtJAZs in Clade4. Six JAZ proteins were in Clade5 and 11 members in Clade6, and both groups had only AtJAZs, SlJAZs, and StJAZs. Nineteen protein members were in Clade 7, and it contains five species of JAZ. In addition, we found StJAZ preferred to cluster with AtJAZ and SlJAZ in each group. These results indicate that StJAZ had a distant evolutionary relationship with physcomitrella patens and rice, and it is more closely related to *Arabidopsis thaliana* and tomato.

In the same way, an unrooted NJ phylogenetic tree was constructed from 59 MYC proteins (including 12 StMYCs, 16 AtMYCs, 12 PpMYCs, eight OsMYCs, and 11 SlMYCs). The *MYC* genes could be divided into five major subfamilies (Figure 2B). Surprisingly, each subfamily had similar numbers of MYC proteins. It is noteworthy that members from Clade2, Clade3, Clade4, and Clade5 had a variety of MYC members from both dicot and monocots plants; Clade1 looked to be a dicot-only MYC clade in this analysis. Clade5 does not contain the MYC protein of potatoes and tomatoes, and the evolutionary distance between Clade5 and other clades is large. These results show that the *MYC* gene may branch in dicots during evolution.

### 2.3. Gene Structure and Protein Motif Analysis of StJAZ and StMYC

**Figure 3:**
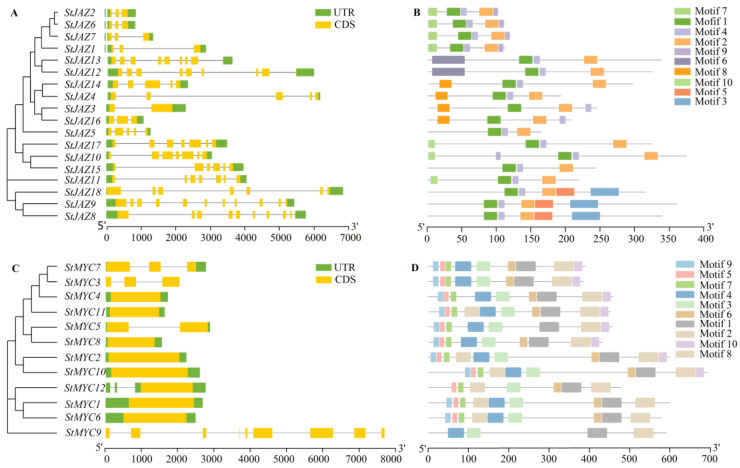
*StJAZ* and *StMYC* genes Structure and motif. (**A**) The gene structure analysis of *StJAZ*; (**B**) The motif composition in potato JAZ proteins; (**C**) The exon–intron structure of *StMYC*; (**D**) The motif composition of StMYC proteins. UTRs are represented by green rectangles, exons by yellow rectangles, and introns by black lines.

Comparing the exon/intron structures and motif composition of *StJAZ* gene families revealed that individuals belonging to the same subfamily shared comparable intron/exon patterns and motif composition. As shown in Figure 3A, StJAZ1, StJAZ2, StJAZ6, and StJAZ7 are all in Clade2, and they all contain three exons. In addition, they had the same motifs and variety (Figure 3B). Surprisingly, StJAZ3, StJAZ4, StJAZ14, and StJAZ16 are all in Clade1, and they had the same motifs and variety, but the number of exons is different. The investigation of the *StMYC* gene’s exon/intron structures and protein motif component analysis showed that each gene had a similar motif type and number, except for StMYC9 (Figure 3C,D). Most genes had only one exon; *StMYC3*, *StMYC5*, and *StMYC7* had 2–3 exons; and *StMYC9* contained the most exons.

Overall, in the StJAZ and StMYC families, members of the same subfamily have comparable intron/exon patterns and motif components. For the StJAZ family, different subfamily members are different in exons, motif type, and number. For the StMYC family, members of the different subfamilies have similar motifs and variety, but the exon/intron structure was slightly different. The results show that the *JAZ* gene family and *MYC* gene family may have undergone different evolutionary selection events.

### 2.4. Promoter Cis-Acting Analysis of StJAZ Genes

**Figure 4:**
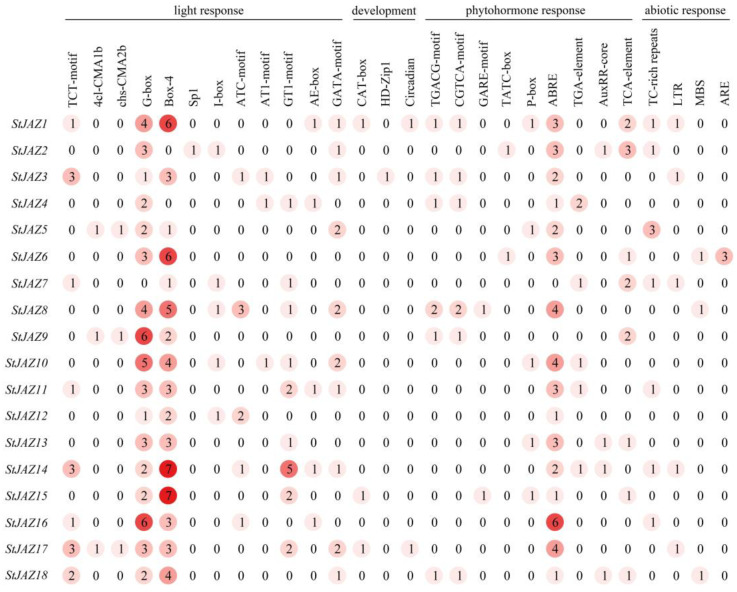
*StJAZ* genes promoter cis-acting elements. The different colors and numbers in the circle represent the number of cis-acting elements in the promoter area of the StJAZs. As the number in the circle increases, the color of the circle becomes darker.

Cis elements present upstream of genes are important for revealing gene function. Promoter analysis of *StJAZ* genes showed that 28 elements were found (Figure 4), which were divided into four broad categories: development, light response, phytohormone response, and abiotic response elements. Most *StJAZ* genes contain a plentiful of core promoter elements, such as G-box, Box-4, GATA-motif, GT1-motif, ABRE, and TCA-element, which are present in light response or phytohormone response. Moreover, the GAT-box, Circadian elements associated with the development, and the TC-rich repeats, LTR, and MBS elements associated with abiotic response were found in some *StJAZs*. These findings revealed that *StJAZ* was involved in the control of potato plant growth and development, as well as its response to abiotic stress.

### 2.5. Interaction Networks of StJAZ and StMYC Proteins

**Figure 5:**
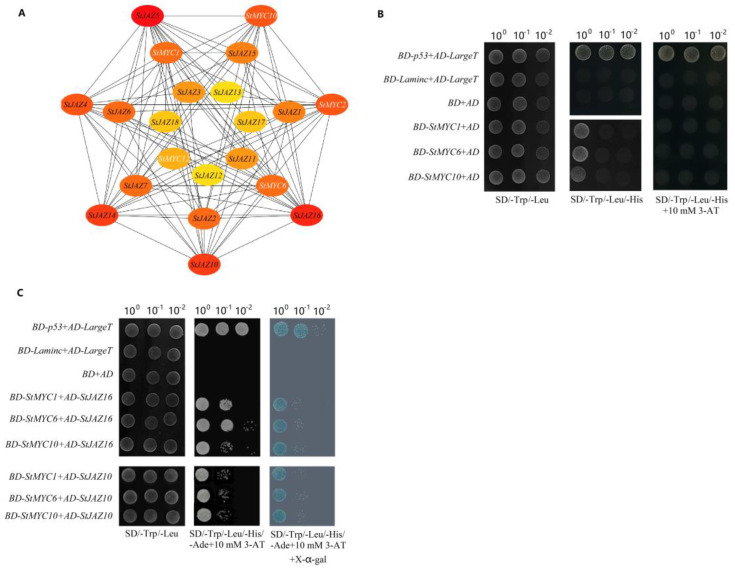
PPI network analysis of *StJAZ* and *StMYC* genes. (**A**) The PPI network of StJAZs and StMYCs; (**B**) The result of self-activation detection; (**C**) Yeast two−hybrid interactions between StJAZ proteins and StMYCs.

To find more StMYC transcription factors that can interact with StJAZ proteins, a PPI network based on StJAZ and StMYC proteins was constructed. Protein interaction prediction results showed that a total of five StMYC proteins interacted with 16 StJAZ proteins. In addition, *StMYC2* can interact with StMYC10 and some StJAZ proteins can interact with each other (Figure 5A).

StMYC1, StMYC6, StMYC10, StJAZ10, and StJAZ16 were selected to verify that the PPI network was correct. The self-activation detection of the three MYC transcription factors showed that the three transcription factors had different degrees of self-activation. Autoactivation was completely inhibited when 10 mM 3-AT was applied in the medium of SD/-Trp/-Leu/-His (Figure 5B). The yeast two-hybrid assay results showed that StMYC1, StMYC6, or StMYC10 could all interact with StJAZ10 or StJAZ16 (Figure 5C). This is consistent with the results of PPI network. These results indicated that StJAZs may interact with some MYC TFs to take part in the associated regulatory network.

### 2.6. Expression Analysis of StJAZ and StMYC Genes with Interactions and Their Responses to Abiotic Stress

**Figure 6:**
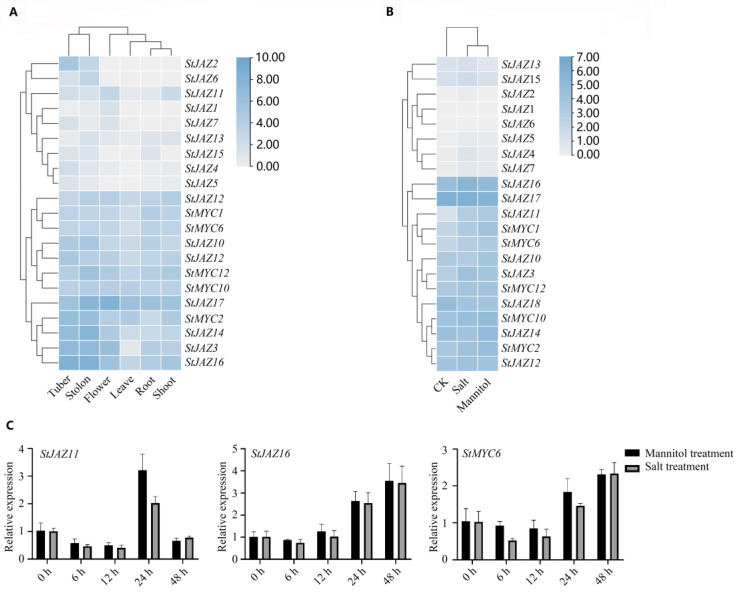
Expression patterns of *StJAZ* and *StMYC* genes with interactions and their responses to abiotic stress. (**A**) *StJAZ* and *StMYC* expression patterns in various tissues. (**B**) Expression patterns of *StJAZs* and *StMYCs* in different treatments. (**C**) Analysis of the expression of *StJAZ11*, *StJAZ16*, and *StMYC6* by quantitative real-time (qRT)-PCR.

To understand the tissue-specific expression patterns and to investigate the roles of *StJAZ* and *StMYC* genes with interactions in response to environmental stresses, the transcript data from relevant organizations are mainly from NCBI, including root, shoot, tuber, leave, flower, salt treatment (150 mM, 24 h) and mannitol treatment (260 mM, 24 h). Two heatmaps were created based on the transcript data of 16 *StJAZ* and five *StMYC* genes (Figure 6A,B). As shown in Figure 6A, six *StJAZ* and five *StMYC* genes are expressed in all tissues, and other genes exhibited distinct tissue-specific expression patterns. As shown in Figure 6B, a total of six *StJAZ* genes (*StJAZ1*, *StJAZ2*, *StJAZ4*, *StJAZ5*, *StJAZ6*, and *StJAZ7*) is hardly expressed in all treatments; other *StJAZ* and *StMYC* genes are expressed in all samples (FPKM > 1 and |log^2FC^| > 1). In addition, some gene’s expression levels showed some changes in different stresses compared to controls. Under the stress of salt and drought, *StJAZ3*, *StJAZ11*, *StJAZ16*, and *StMYC1*, *StMYC6*, and *StMYC12* genes were upregulated, and *StJAZ18* genes were downregulated.

Based on the PPI network and transcriptome data, 2 *StJAZ* genes (*StJAZ11*, *StJAZ16*), and *StMYC6* genes were chosen for further qRT-PCR study under various abiotic conditions. Salt or mannitol stress increased the expression level of the chosen genes at 24 h, which is consistent with the RNA-Seq results.

As shown in Figure 6C, salt or mannitol stress increased the expression level of the chosen genes at 24 h, which is similar to the RNA-Seq data. StJAZ11 expression displayed a comparable down-up-down pattern in salt and mannitol treatments, peaking after 24 h of treatment before beginning a rapid drop. StJAZ16 expression was a down-up pattern in different treatments, with the lowest expression after 6 h of treatment, and then began to rise. StMYC6 and StJAZ11 had similar expression patterns before 24 h of treatment, but their expression trends differed at 48 h after treatment. StMYC6 and StJAZ11 had similar expression patterns too, and StMYC6 expression is lowest later than StJAZ16. These results showed that StJAZ11 and StJAZ16 may interact with StMYC6 to participate in potato response stress.

## 3. Discussion

*JAZ* is a family of genes unique to plants that play a prominent role in many physiological processes, including plant growth and stress response, primarily by modulating JA signaling [20]. According to studies, the JAZ protein mainly regulates the physiological processes of plants by binding to or releasing the MYC transcription factor [21]. However, there have been few studies on potato *JAZ* and *MYC* genes. Therefore, in this work, we identified StJAZ and StMYC family members of potatoes, analyzed their sequences, structures, and interactions between family members, and explored some members’ responses to abiotic stress. Other species have been identified to have members of the JAZ and the MYC family. In this study, we identified a total of 18 StJAZ family members and 12 StMYC family members. Previous studies have found 13, 16, 36, and 26 JAZ members in Arabidopsis thaliana ([22], maize [23], turnip [24], and tomato [25], respectively. For the MYC gene, 27 members were identified in wheat [26], 14 in camellia [27], and 17 in cabbage [28]. It can be seen that, whether it is the MYC family or the JAZ family, the number of members varies among different species.

Research has revealed that the JAZ protein can bind to a variety of MYC TFs to affect the JA reaction. In this study, we found that five MYC transcription factors in potatoes interact with multiple JAZ proteins by constructing a PPI network between two gene families, and yeast two-hybrid assay results prove the authenticity of the PPI network. *StMYC6* is a homologous gene of *Arabidopsis thaliana* AtMYC2 and AtMYC4 in potatoes, and *StMYC2* is a homologous gene of AtMYC3, both of which are in the PPI network. This is consistent with Fernández-Calvo et al. [15] and Schweizer et al.’s [16]. findings. However, whether *StMYC1*, *StMYC10*, and *StMYC12* interact with the JAZ protein in other species is unknown. Our research indicates for the first time that there may be more MYC transcription factors in potatoes involved in signal transduction of JA than in Arabidopsis.

JAZ protein is important for plants to resist environmental stresses [10]. This study used transcriptome data and qRT-PCR analysis to find that some JAZ proteins and *MYC* genes were expressed differently under drought and salt stress, which stays in step with the findings of Wu et al. and Fu et al. [29,30]. StJAZ11 showed an up and down mode in this treatment, and the level of expression was lower than 0 h at 24 h after treatment, which indicates that StJAZ11 may be involved in the stress reaction. StJAZ16 expression showed an overall upward trend, revealing that StJAZ16 may contribute to potato resistance to abiotic stress. In addition, we found that the patterns of expression of StJAZ11 and StJAZ16 in response to drought and salt stress are inconsistent, suggesting that it is possible that different JAZ proteins can participate in different pathways in response to stress. The expression profile of StMYC6 is comparable to StJAZ16, explaining that StJAZ16 may cooperate with StMYC6 to participate in potato response stress. These conjectures also require subsequent functional validation of genes to reveal their role in JA signaling.

## 4. Materials and Methods

### 4.1. Identification of the Potato JAZs and MYCs

To search for the *JAZ* gene in the potato genome, Markov Model (HMM) files for the ZIM domain (PF06200) and JAS domain (PF09425) in the Pfam protein family database (http://pfam.sanger.ac.uk/, accessed on 7 October 2022) were downloaded. In HMMER 3.0 software, the previous two HMM profiles are used to search the potato protein database for target hits in ZIM and JAS domains. The candidate JAZ proteins with E-values < 1.0 × 10^−5^ were selected. We used the same method to find b HLH_M-YC_N domain (PF14215) and HLH domain (PF00010) to identify MYC family members. The online tool ExPASy (http://www.expasy.org, accessed on 10 October 2022) was used to analyze the molecular weight (MW) and isoelectric point (pI) of each JAZ protein and MYC TF.

The WoLF PSORT (https://wolfpsort.hgc.jp/, accessed on 12 October 2022) is used to predict the subcellular localization of each gene. The protein sequences of related genes are sorted into txt files and placed on the website, and the predicted positioning with the highest score is selected as the target.

### 4.2. Chromosomal Distribution of StJAZs and StMYCs

Based on the chromosomal position provided in the annotated genome, the StJAZs and StMYCs were separately mapped to the potato chromosome and visualized using TBtools software.

### 4.3. Analysis of Conserved Motif and Gene Structure

Multiple protein sequence alignments were created for StJAZs and StMYCs proteins, respectively, using the default parameter settings of the MEGA X software and the MUSCLE method [31]. The gene structure is performed using TBtools software based on genomic information. MEME (http://meme-suite.org/, accessed on 15 October 2022) and TBtools [32] were used to identify and optimize conserved motifs in potato StJAZs and StMYCs proteins, respectively.

### 4.4. Phylogenetic Analysis

The Ensembl Plants database (https://plants.ensembl.org/index.html, accessed on 21 October 2022) was used to obtain genome sequences and annotation files for *Arabidopsis thaliana*, rice (*Oryza sativa* L.), physcomitrella patens *(Physcomitrium patens)*, tomato (*Solanum lycopersicum*) and potato.

Using the default parameter settings in MEGA X software, the StJAZs and StMYCs protein sequences of the potato, tomato, Arabidopsis, physcomitrella patens, and rice are aligned using the MUSCLE method. Based on this result, a neighborhood connection (NJ) phylogenetic tree with 1000 bootstrap values was constructed. Additionally, Evolview (http://www.evolgenius.info/evolview, accessed on 28 October 2022) was used to visualize the phylogenetic tree.

### 4.5. Analysis of StJAZs Gene Promoter Cis-Acting Element

The upstream 1500 bp of the *StJAZs* gene is obtained from Ensembl Plants as the initiation sequence, and PlantCARE [33] was used to analyze the cis-acting elements of promoters. It was finally visualized using TBtools.

### 4.6. StJAZ and StMYC PPI Network Analysis

Version 11.0 of the STRING database is used to create the PPI networks of StJAZs and StMYCs [34], and the network map was beautified by Cytoscape 3.91 software [35].

### 4.7. Self-Activation Detection and Yeast Two-Hybrid Assay

StMYC1, StMYC6, and StMYC10 were connected to the *Eco*RI and *Bam*HI sites of pGBKT7 (BD). The resulting plasmid was transformed into AH109 yeast strain together with pGADT7 (AD). The BD-53 and AD-LargeT were used as positive controls. The BD-lamins and AD-LargeT, BD, and AD vectors were used as the negative controls. The transformants were inoculated on SD/-Trp/-Leu and SD/-Trp/-Leu/-His plates and incubated at 30 °C for 3–5 days and screened with different concentrations of 3-AT on SD/-Trp/-Leu/-His plates. The primers used for self-activation detection are listed in Supplementary Table S3.

The EcoRI and BamHI sites of AD were ligated with StJAZ10 and StJAZ16. The BD-StMYC1, BD-StMYC6, and BD-StMYC10 were transformed into AH109 yeast strain, together with AD-StJAZ10 and AD-StJAZ16. The positive and negative controls were consistent with the self-activation detection. The transformants were inoculated on SD/-Trp/-Leu, and SD/-Trp/-Leu/-His/-Ade with 3-AT plates at 30 °C for 3–5 days, and X-α-gal is used to detect galactoside activity. The primers used for self-activation detection are listed in Supplementary Table S3.

### 4.8. Expression Analysis from RNA-Seq Data

To examine the patterns of the *StJAZ* and *StMYC* gene expression in various tissues (root, stem, leaf, flower, stolon, tuber) and under various abiotic stresses (150 mmol/L NaCl and 260 μmol/L mannitol for 24 h), the Illumina RNA-seq data were collected from the NCBI (Accession number: SRA029323).

### 4.9. Plant Treatments and RT-qPCR Analysis of StJAZ and StMYC Genes of Potatoes under Abiotic Stress

Potato Cultivar Desiree was used in this study. MS medium with pH 5.8, 3% sucrose and 0.8% agar was used for growth of abiotically stressed potatoes in an external environment at 22 °C, with a photoperiod of 16 h/8 h (light/dark). After 1 month, potato plantlets were subjected to drought and salt stress, including salt (150 mM NaCl) and simulated drought (260 mM mannitol) treatments. Plantlets that were not treated served as the control (CK). After treatment, the control and treated plantlets were harvested at 0, 6, 12, 24, and 48 h. They were then refrigerated at −80 °C.

The TaKaRa MiniBEST Plant RNA Extraction Kit’s instructions were followed, and the total RNA extraction was performed on the treated plants. The SYBR Premix Ex Taq (Takara) kit was used to conduct qRT-PCR studies on the cDNA samples. As an internal control, the *SteIF-5A-4* gene (PGSC0003DMT400068977) was used. Each treatment contains three biological replicates (each containing three plants) and three technical replicates. To determine the relative expression level of a gene, the 2^–ΔΔCt^ method was applied [36]. In Supplementary Table S3, primers used for qRT-PCR analysis are listed.

## 5. Conclusions

This study provides the first comprehensive and systematic analysis of information about the *JAZ* and *MYC* gene families in potatoes. A total of 18 *StJAZ* and 12 *StMYC* genes in the potato genome were detected. The *JAZ* and *MYC* gene families have undergone different evolutionary selection events based on genome-wide investigation and comprehensive analysis. Sixteen StJAZ proteins, based on the PPI network and yeast two-hybrid assay results, could interact with five StMYC transcription factors, which provides new insights for the operation mechanism of StJAZ and StMYC in JA signal response. The study of StJAZ16 and StMYC6 revealed that they were involved in the response of potatoes to abiotic stress. This study enriches the understanding of potato JAZ genes and MYC TFs and establishes the framework for future research on the functions of StJAZs and StMYCs in potatoes, which opened up a new research direction for the genetic enhancement of potato abiotic stress resistance.

## Figures and Tables

**Table 1:** Characteristics of 18 family members of the *JAZ* gene in *Solanum tuberosum*.

Gene ID	CDS Lengths (bp)	Protein Lengths (aa)	PI	MW	Localization
*StJAZ1*	351	117	9.3	13,350.27	Nucleus
*StJAZ2*	321	107	9.81	12,530.24	Nucleus
*StJAZ3*	765	255	9.24	28,431.98	Nucleus
*StJAZ4*	604	201	9.3	22,397.20	Nucleus
*StJAZ5*	531	177	9.01	19,473.24	Nucleus
*StJAZ6*	348	116	9.74	13,198.17	Nucleus
*StJAZ7*	375	125	9.17	14,341.22	Nucleus
*StJAZ8*	1023	341	5.11	38,583.98	Nucleus
*StJAZ9*	1128	376	4.93	40,849.43	Nucleus
*StJAZ10*	1173	391	9.43	41,118.75	Nucleus
*StJAZ11*	687	229	6.4	26,127.31	Nucleus
*StJAZ12*	1020	340	8.23	37,486.27	Nucleus
*StJAZ13*	984	328	8.45	39,257.33	Nucleus
*StJAZ14*	930	310	8.51	33,698.76	Nucleus
*StJAZ15*	777	259	9.71	27,460.62	Nucleus
*StJAZ16*	648	216	8.84	23,905.17	Nucleus
*StJAZ17*	1017	339	8.93	35,760.7	Nucleus
*StJAZ18*	987	329	6.13	34,998.6	Nucleus

**Table 2:** Characteristics of 12 family members of the *MYC* gene in *Solanum tuberosum*.

Gene ID	CDS Lengths (bp)	Protein Lengths (aa)	PI	MW	Localization
*StMYC1*	1797	599	6.86	66,369.87	Nucleus
*StMYC2*	1953	651	5.92	65,258.62	Nucleus
*StMYC3*	432	144	6.03	44,083.76	Nucleus
*StMYC4*	1374	458	5.99	50,898.24	Nucleus
*StMYC5*	1371	457	6.22	51,791.64	Nucleus
*StMYC6*	1737	579	7.61	64,285.91	Nucleus
*StMYC7*	1224	408	5.88	44,514.29	Nucleus
*StMYC8*	1296	432	6.71	48,790.06	Nucleus
*StMYC9*	2064	688	5.81	64,760.08	Nucleus
*StMYC10*	2109	703	5.47	75,939.94	Nucleus
*StMYC11*	1359	453	5.61	49,967.36	Nucleus
*StMYC12*	1434	478	6.59	52,903.87	Nucleus

## Data Availability

Not applicable.

## Supplementary Materials

The following supporting information can be downloaded at: https://www.mdpi.com/article/10.3390/ijms24076706/s1.
